# The Effect of the Underlying Distribution in Hurst Exponent Estimation

**DOI:** 10.1371/journal.pone.0127824

**Published:** 2015-05-28

**Authors:** Miguel Ángel Sánchez, Juan E. Trinidad, José García, Manuel Fernández

**Affiliations:** 1 Department of Mathematics, Universidad de Almería, Almería, Spain; 2 Department of Economics, Universidad de Almería, Almería, Spain; 3 University Centre of Defence at the Spanish Air Force Academy, MDE-UPCT, Santiago de la Ribera, Murcia, Spain; East China University of Science and Technology, CHINA

## Abstract

In this paper, a heavy-tailed distribution approach is considered in order to explore the behavior of actual financial time series. We show that this kind of distribution allows to properly fit the empirical distribution of the stocks from S&P500 index. In addition to that, we explain in detail why the underlying distribution of the random process under study should be taken into account before using its self-similarity exponent as a reliable tool to state whether that financial series displays long-range dependence or not. Finally, we show that, under this model, no stocks from S&P500 index show persistent memory, whereas some of them do present anti-persistent memory and most of them present no memory at all.

## Introduction

A classic topic in finance is about the behavior of stock market prices. This issue mainly covers two areas: the first one is related with the idea of market efficiency, namely, if stock prices are independent random variables, whereas the another is about the underlying distribution in market prices. Indeed, both topics are connected.

The foundations of the so-called Efficient Market Hypothesis (EMH, herein) were first collected by Cootner in [1]. Such a hypothesis implies that stock returns become independent and identically distributed (i.i.d., herein) random variables.

Sometimes it is assumed that the underlying distribution for stock market (log returns) is the normal one. However, the application of normal distributions to model stock returns leads to an extreme simplification of reality. In this way, the first to show that empirical data exhibits more kurtosis and variance than normal distributions may report was Fama in [2] (1965). Since then several distributions have been contributed to model stock market returns. For instance, both normal and log normal distributions have been applied in financial literature as a good fit for financial series, mainly since their parameter estimation becomes relatively easy, being the (log-) normal one quite suitable for modeling purposes. Nevertheless, some works have reported empirical evidence against the convenience of their use in finance [3].

In addition to that, the Student distribution becomes another widely used model in literature. This presents fatter tails than a normal distribution though it does not result helpful for skewness. Indeed, recall that due to Central Limit Theorem (CLT), the addition of Student random variables leads to a normal one.

Another extended practice consists of the use of more flexible parametric distributions, namely, families of distributions which include many other probability distributions as particular cases. One of the most popular among them is the so-called stable distribution, which was first introduced by Mandelbrot in [4]. Moreover, some variations for stable distributions were contributed in [5–12], to quote some of them. Thus, note that Generalized CLT states that the sum of i.i.d. stable variables remains also stable. Nevertheless, their variance may be equal to infinite, that constitutes the key reason for which such a parametric family has been mostly rejected in financial literature to model asset returns (see [6, 8, 13]).

On the other hand, both Pearson distribution (see [14, 15]) as well as logistic distribution constitute non-standard alternatives to model stock returns [16, 17]. Further, the distribution of (log) stock returns has been understood also as a mixture of distributions (see [18, 19]). In this way, (exponential) generalized beta and generalized *t* constitute parametric distributions which allow a mixture interpretation.

Some general aspects about EMH were questioned by pioneer B. Mandelbrot. In fact, in [4] he concluded that stock prices could be fitted by means of a fractional Brownian motion, what is equivalent to state that prices exhibit long-memory. However, to determine whether market is efficient is not a simple matter: a wide amount of works support the EMH (see [20–24]), whereas some papers provide arguments against it (see [25–27]).

The concept of long-memory has been related to EMH in some recent works [28, 29]. In this way, scaling patterns have been increasingly explored for financial markets: in such a context, [30–34] constitute a sample of quite interesting contributions. Recall that such a connection was mainly introduced in [30], where it was considered that market agents may be essentially distinguished by the frequency at which they operate in markets.

Moreover, note that this also provides a link to the so-called Fractal Market Hypothesis (FMH, herein), which is not necessarily in conflict with EMH. Firstly, FMH emphasizes the impact of information and expectations on the investor behavior [35, 36]. In classical finance theory, information is treated as a generic item, so EMH implies that all types of information impact investors (also generic) in a similar way. In addition to that, FMH states that information is valued according to each investor’s horizon. Hence, information has a different value for each investors group as well as each of them has its own trading rules and strategies. Indeed, while traders are only focused on short terms, investors are mainly interested in long-term investments. In fact, both EMH and Capital Asset Pricing Model (CAPM) seem to work fine provided that markets are considered to be stable, except during panics or crisis when correlations increase [37].

This is not unexpected, since EMH, Arbitrage Pricing Theory (APT) and CAPM constitute balanced models which do not work properly under turbulence. On the other hand, FMH is based on liquidity, which throws smooth pricing market processes, making it more stable. Therefore, the existence of investors having different horizons leads to a stable market evolution, though market may become unstable when one horizon becomes dominant since liquidity ceases. In this way, FMH predicts that critical events are connected to dominating investment horizons. Moreover, FMH is usually connected to either fractality or multifractality patterns on stock market processes (see [38]). On the other hand, in [39] it was shown throughout Continuous Wavelet Transform analysis (CWT) that short investment horizons dominate markets during financial crisis. Overall, scaling analysis provides a suitable approach to check the relative impact of such heterogeneous agents on price movements.

We would also like to point out that several authors suggest that EMH could be understood as the result of interactions among these agents which do indeed characterize a mature market [29, 40–44]. Thus, they assume that such market features should be quantified by means of their corresponding self-similarity exponents, so they analyze scaling behavior patterns in order to characterize markets. Overall, under such considerations, they state that developed market price series usually throws only short memory or no memory at all (namely, *H* ≤ 0.5), whereas emerging markets do exhibit long-memory properties.

There is a wide range of approaches in scientific literature to deal with Hurst exponent estimation. Among them, both R/S analysis (introduced in finance by Mandelbrot and Wallis in [45]) and Detrended Fluctuation Analysis (first appeared in [20]) are, maybe, the most widely applied procedures for this purpose. Nevertheless, inconveniences for both methodologies are well-known. For instance, in [27, 46, 47] it was proved that the self-similarity exponent estimation through those classical models presents important deviation, especially if the length of time series is not large enough. Further, R/S analysis implicitly assumes that stock market prices follow a fractional Brownian motion.

Alternative self-similarity index estimations have been contributed afterwards, including Multifractal Detrended Fluctuation Analysis (MF-DFA) [48], Lyapunov exponent [49, 50], Hudak’s Semiparametric Method (GPH) [51], Quasi Maximum Likelihood analysis (QML) [52], Centered Moving Average (CMA) [53], Generalized Hurst Exponent (GHE) [54] and recently, Geometric Method-based procedures [55] and fractal dimension algorithms [56], to quote some of them.

Furthermore, DMA approach was first developed in [53, 57] to deal with long-range correlations for non-stationary fluctuating signals and revisited afterwards in [58]. Such an approach presents an advantage over DFA procedure, since DMA allows to estimate correlation properties for nonstationary signals without requiring additional assumptions over the underlying stochastic process, including the type of trends [59]. We would like also to point out that its implementation does not performs a division of the series into boxes, so scaling patterns are explored through moving average. This fact makes DMA algorithm quite efficient from a computational point of view. Interesting applications of DMA approach could be found in literature, including WTI crude oil futures market [60] and [61], where it was compared the performance of four estimators (FA [62], DFA, and two versions of DMA using backward (BDMA) and centered (CDMA) means, respectively). Authors proved there that both CDMA and DFA do show a similar performance. Moreover, in [63], DMA procedure was extended in order to properly deal with multifractal series through MF-DMA. Thus, the authors contributed empirical evidence that for multifractal time series, MF-DMA performs better than MF-DFA. On the other hand, a novel kind of approaches are raising in recent papers in order to test for long-range power law cross-correlations among nonstationary time series. In this way, let us quote among them, Detrended Cross-Correlation Analysis (DCCA) [64–66], Multifractal Detrended Cross-Correlation Analysis (MF-DXA, introduced in [67]), or recently, Multifractal Detrending Moving-Average Cross-Correlation Analysis (MF-X-DMA) [68].

Unfortunately, most of the approaches that are valid to calculate the self-similarity exponent for fractional Brownian motion based series do not work properly whether the underlying distribution is no longer the normal one. In fact, the interpretation of results in such cases may be far away from reality (see [69, 70] for Lévy stable motions case). Thus, the main goal in this paper is to show that *elevated two-sided power* distributions (ETSP, herein) could properly fit the evolution of financial time series. In addition to that, we explain also in the present work why a preliminary study regarding the underlying distribution of such series results quite useful to appropriately test for long-range dependence. The interpretation of the self-similarity exponent through this novel approach becomes completely different from the classical one.

## Materials and Methods

### Self-similar processes

Let (*X*, 𝓐, *P*) be a probability space, with *t* ∈ [0, ∞) being time. Recall that **X** = {*X*(*t*, *ω*):*t* ≥ 0} is a random process or a random function from [0, ∞)×Ω to ℝ, if *X*(*t*, *ω*) is a random variable for all *t* ≥ 0 and all *ω* ∈ Ω, where *ω* belongs to a sample space Ω. Thus, we consider **X** as defining a sample function *t* ↦ *X*(*t*, *ω*) for all *ω* ∈ Ω. In this way, note that the points of Ω parametrize the functions **X**:[0, ∞) × Ω → ℝ, and *P* is a probability measure on this class of functions.

Let *X*(*t*, *ω*) and *Y*(*t*, *ω*) be two random functions. The notation *X*(*t*, *ω*) ∼ *Y*(*t*, *ω*) means that they have the same finite joint distribution functions. A random process **X** is said to be self-similar [71] if there exists a parameter *H* > 0 such that

$$\begin{array}{c} X\left(a\;t,\omega \right)∼a^{H}\;X\left(t,\omega \right), \end{array}$$ (1)

for all *a* > 0 and *t* ≥ 0. Equivalently, **X** is also called a *H*-self-similar random process, and the parameter *H* is its self-similarity index or exponent. Thus, if *X*(*t*, *ω*) denotes space (with *t* being time), then Eq (1) establishes that every change of time scale *a* > 0 corresponds to a change of space scale *a* ^*H*^. Moreover, the bigger *H*, the more drastic is the change of the space variable. Additionally, Eq (1) implies also a scale-invariance of the finite dimensional distribution of the random process **X**.

A wide class of processes are self-similar. They include (fractional) Brownian motions as well as their generalizations, namely, (fractional) Lévy stable motions. In the fractional Brownian motion case, the self-similarity index *H* is also called Hurst exponent. Some authors also use the name Hurst exponent as a reference to the self-similarity index for any self-similar random process.

### Generalized Hurst Exponent

The original procedure contributed by Hurst in [72] was generalized afterwards in [54, 73] to test for long-range correlations of time series. This approach is based on the *q*th-order moments of the distribution of the increments of the corresponding stochastic process *X*(*t*) which describes the evolution of the time series. According to that, the scaling properties of a (financial) time series are explored through a *q*th-order statistic we denote by *K*_*q*_(*τ*), which is given as follows (see [74]):

$$\begin{array}{c} K_{q}\left(\tau \right)=\sum_{t=0}^{T-\tau }\frac{{\left|X(t+\tau )-X(t)\right|}^{q}}{T-\tau +1}, \end{array}$$

where *T* is the length of the time series *X*(*t*) (namely, the observation period) and *τ* ∈ {1, …, *τ*_max_} is the number of days considered. Further, it is verified that the statistical properties of *X*(*t*) scale with the resolution of the time window.

Notice also that the next alternative expression for *K*_*q*_(*τ*) could also be found in the literature (see [28, 69, 74]):

$$\begin{array}{c} K_{q}\left(\tau \right)=\frac{\left\langle {\left|X(t+\tau )-X(t)\right|}^{q}\right\rangle }{\left\langle {\left|X(t)\right|}^{q}\right\rangle }, \end{array}$$

where ⟨ ⋅ ⟩ denotes the sample average over the corresponding time window. Overall, the statistic *K*_*q*_(*τ*) scales following a power-law like the next one:

$$\begin{array}{c} K_{q}\left(\tau \right)\propto \tau^{q\;H(q)}. \end{array}$$ (2)

Thus, the scaling behavior of *K*_*q*_(*τ*) allows to find the so-called *generalized Hurst exponent* (GHE, herein) [75]. Observe that all the information about scaling properties of *X*(*t*) is contained in its scaling index *H*(*q*). This makes the GHE based analysis quite simple to test for scaling properties of time series.

As well as it happens with other tools to test for scaling properties of time series (see for example, MF-DFA [76], MF-DMA [63] and FD4 [77]), GHE provides a valid procedure to deal with multi-fractal (also multi-scaling) processes, namely, those ones in which the scaling exponent *H*(*q*) depends on each moment *q*. In such case, different exponents characterize the scaling properties of different *q*th-moments for the underlying distribution [78, 79]. According [28], the nonlinear properties of *q* *H*(*q*) vs. *q* (or equivalently, the linear patterns of *H*(*q*) vs. *q*) throw empirical evidence against the classical models proposed to fit actual (financial) time series, including (fractional) Brownian motions as well as (fractional) Lévy stable motions.

On the other hand, it is quite natural to apply GHE to properly study unifractal processes. Thus, scaling properties of such processes is uniquely determined through a single constant *H* which agrees with the self-similarity exponent (resp. Hurst exponent) of the time series. Note that in this case, the plot for *q* *H*(*q*) vs. *q* provides a straight line (equivalently, *H*(*q*) = *H* for each *q*th-order moment, that is, the self-similarity index remains constant).

In particular, if *q* = 1, then GHE provides a valid tool to analyze the scaling properties of the absolute deviations of the process under consideration. Thus, the quantity *H*(1) is expected to be close to the original Hurst exponent.

It is worth mentioning that in [74], it was shown that among all procedures considered to test for long-range correlation properties, only GHE (*q* = 1) and MF-DFA (*q* = 1) approaches could be used to properly calculate the self-similarity index for heavy-tailed distributions (see [74]). In addition to that, it was reported in such work that GHE (*q* = 1) performs much better than MF-DFA (*q* = 1). According to that, in this paper we will use GHE with *q* = 1 for Hurst exponent calculation purposes.

Note that it could be also possible to consider *q* = 2 in Eq (2) for long-range dependence detection purposes. Indeed, recall that in such case, *K*_2_(*τ*) is related with the scaling of the autocorrelation function of the time series increments and it is also connected to the power spectrum. Indeed, such an autocorrelation function has the following expression: *α*(*t*, *τ*) = ⟨*X*(*t*+*τ*) *X*(*t*)⟩.

Hence, the empirical estimation of *H*(2) from *K*_2_(*τ*) ∝ *τ* ^2 *H*(2)^ is somehow equivalent to estimate the Hurst exponent (resp. the self-similarity exponent) *H* by means of any other classical procedure appeared in literature, such as R/S or MF-DFA.

We would like also to point out that GHE was revisited in [80] to test for scaling properties of financial time series. Further, though some recent works reported empirical evidence about multi-fractal behavior in financial time series, it seems that the source of multifractality has not been completely understood yet (see, for instance, [81, 82]).

### The ETSP distribution

In this paper, we consider Elevated Two-Sided Power distributions. Our purpose is double: first, we will show that they allow to fit properly actual financial time series. Moreover, they also provide a suitable class of distributions valid to point out that previous information about the series under study should be taken into account before calculating its self-similarity exponent and concluding whether there exists memory in that series or not. According to that, in this section we will describe slightly ETSP distributions and we will also explain how to generate samples for such distributions.

ETSP distributions were first introduced in [83] as a new kind of heavy-tailed bounded distributions to model financial time series. The probability density function (pdf, herein) for an ETSP with support [0, 1] is given as the following parametric family of piecewise defined functions:

$$\begin{array}{c} g\left(x|\Theta \right)={𝓒}^{-1}\left(\Theta \right)\cdot \;\{\begin{array}{c} \left(\delta +\left(1-\delta \right)\;n\right)\;\left(\lambda +\left(1-\lambda \right)\;m\;{\left(\frac{x}{\theta }\right)}^{m-1}\right),\;\text{if}\;\;0<x\leq \theta ;\\ \left(\lambda +\left(1-\lambda \right)\;m\right)\;\left(\delta +\left(1-\delta \right)\;n\;{\left(\frac{1-x}{1-\theta }\right)}^{n-1}\right)\;\text{if}\;\;\theta <x<1, \end{array} \end{array}$$ (3)

where Θ = {*θ*, *m*, *n*, *λ*, *δ*}. Note that the parameters in Θ provide different information about the distribution they describe. Indeed, *θ* ∈ [0, 1] is the *threshold* parameter, 0 ≤ *λ*, *δ* ≤ 1 are the *elevation* parameters, and *m*, *n* > 0 are the *power* parameters. Further, observe that the normalization constant 𝓒(Θ) may be described in terms of the parameters in Θ as follows:

$$\begin{array}{c} 𝓒(\Theta )=(1-\theta )\;(\lambda +(1-\lambda )\;m)+\theta \;(\delta +(1-\delta )\;n). \end{array}$$

**Fig 1:**
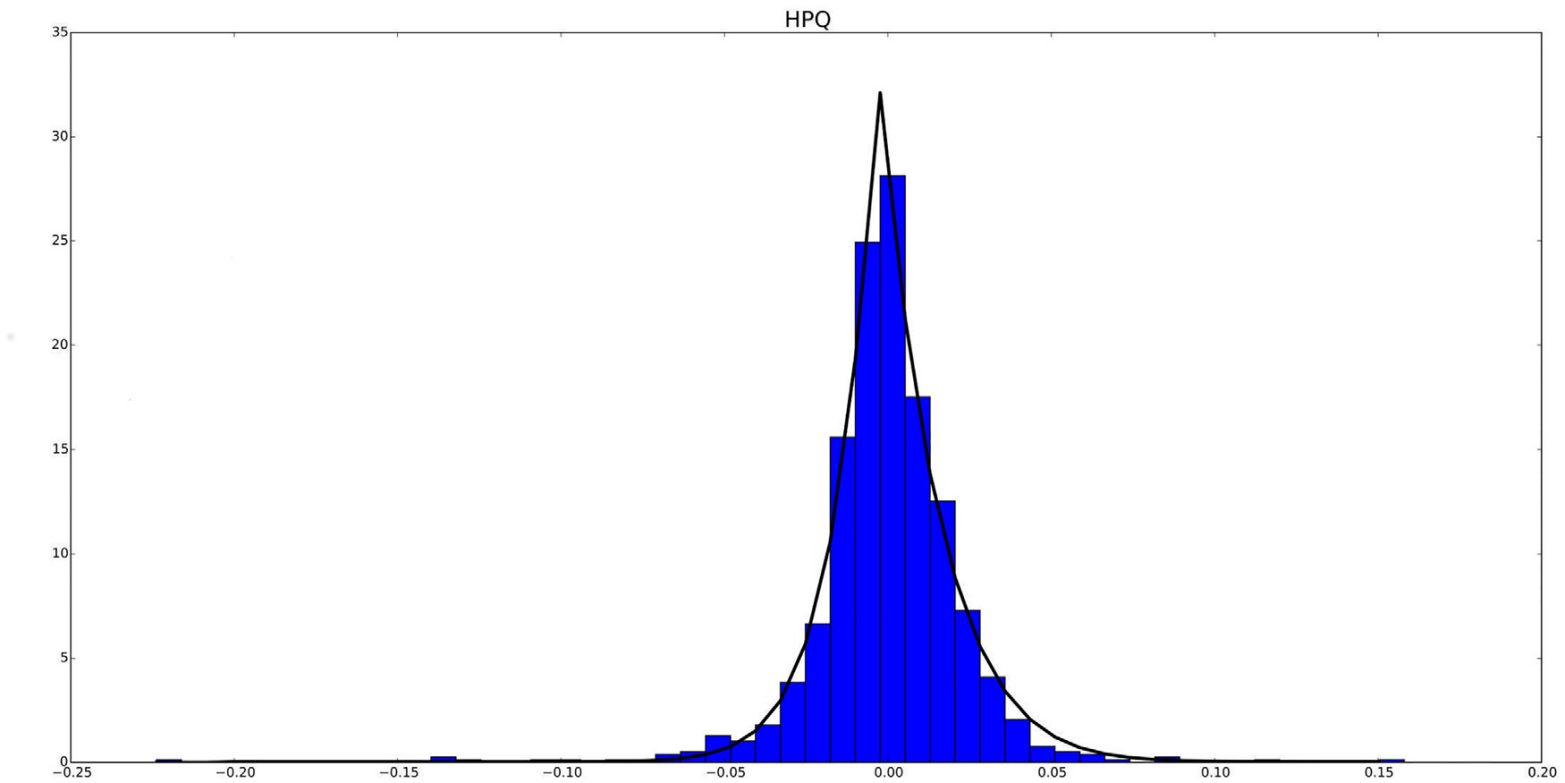
The figure above shows an ETSP pdf plotted over the interval [*a* = −0.19, *b* = 0.21] which properly fits the empirical pdf of a real stock corresponding to Hewlett-Packard Company (HPQ) from S&P500 index. Note that in this case, its corresponding family of parameters was chosen to be as follows: Θ = {*θ* = 0.48, *m* = 13.6, *n* = 13.4, *λ* = 0.0185, *δ* = 0.0170}.

Fig 1 shows an ETSP pdf which properly fits the behavior of the log return of a real stock (HPQ in this case). Additionally, the cumulative distribution function (cdf for short) for Eq (3) has the following expression (see [83]):

$$\begin{array}{c} G\left(x|\Theta \right)=\{\begin{array}{c} x\;\{\frac{\pi (\Theta )}{\theta }\}\;\left\{\lambda +\left(1-\lambda \right)\;{\left(\frac{x}{\theta }\right)}^{m-1}\right\},\;\text{if}\;\;0<x<\theta ;\\ 1-\left(1-x\right)\;\{\frac{1-\pi (\Theta )}{1-\theta }\}\;\left\{\delta +\left(1-\delta \right)\;{\left(\frac{1-x}{1-\theta }\right)}^{n-1}\right\},\;\text{if}\;\;\theta \leq x<1, \end{array} \end{array}$$ (4)

where

$$\begin{array}{c} \pi \left(\Theta \right)=\frac{\theta \;\{\delta +(1-\delta )\;n\}}{\left(1-\theta )\;\{\lambda +(1-\lambda )\;m\}+\theta \;\{\delta +(1-\delta )\;n\right\}}. \end{array}$$

In particular, note that the class of TSP distributions (first introduced in [84]) remains as a particular case of the class of ETSP ones: indeed, it suffices with taking *m* = *n* and *λ* = *δ* = 0. In addition to that, a similar expression to Eq (3) for the description of the ETSP pdf (resp. to Eq (4) for the description of the ETSP cdf) could be obtained in the case of ETSP distributions whose support is a real interval [*a*, *b*]. Accordingly, note that in such case, Θ = {*θ*, *m*, *n*, *λ*, *δ*, *a*, *b*} would be the collection of parameters for this kind of generalized ETSP distributions and that $\frac{X-a}{b-a}$ would be an ETSP distribution in [0, 1].

### Generating ETSP samples

In [83], it was shown how to calculate the quantile functions for an ETSP distribution. That procedure results especially useful for our purposes since it could be used to generate samples for this kind of distributions. In this paper, though, we will apply a faster (preliminary tests showed that this algorithm is about 10 times faster) and more accurate procedure instead of the former algorithm. Next, we describe how to artificially generate ETSP samples through this novel tecnhique. Indeed, let us consider *F*(*θ*) = *G*(*x*∣Θ) as in Eq (4). Hence, if *π* = *F*(*θ*), then the following algorithm could be used:

1. Let *r*_1_, *r*_2_, *r*_3_ be three random (uniform) numbers in [0, 1].
2. If *r*_1_ < *π* and *r*_2_ < *λ*, then return θ*r_3_.
3. If *r*_1_ < *π* and *r*_2_ ≥ *λ*, return $\theta *r_{3}^{\frac{1}{m}}$.
4. If *r*_1_ ≥ *π* and *r*_2_ < *δ*, return 1 − (1 − *θ*)*r_3_.
5. If *r*_1_ ≥ *π* and *r*_2_ ≥ *δ*, return $1-(1-\theta )*r_{3}^{\frac{1}{n}}$.

Note that if we want to generate a sample from an ETSP distribution in an interval [*a*, *b*], we only have to apply the previous algorithm to generate a sample *x* of an ETSP in the interval [0, 1] and then apply the linear transformation *a*+(*b* − *a*) *x*.

## Results

### Linking heavy-tailed distributions to self-similarity exponent

The self-similarity index of both fractional Brownian motions (FBMs onwards) and Lévy stable motions (LSMs, herein) has been widely analyzed in the literature (see, for example, [74]). Due to the Central Limit Theorem and its generalization to stable distributions (see [85]), if financial time series were sums of i.i.d. random variables, then the self-similarity index of such series should yield the type of stable distribution underlying the process. On the other hand, recall that in the FBM case, the self-similarity index is a “measure” to test for long-memory in the series.

In this study, we will focus only on daily changes series. Thus, note that we are not concerned with the intraday process which gives the daily changes, but on the distribution of the daily changes itself. For example, note that if the intraday process were a sum of normal distributed random variables with zero mean and exponentially distributed variances, then the corresponding daily changes would follow a Laplace distribution [86]. In addition to that, note that the Laplace distribution is also related with the TSP distribution (see [84, 87]). Hence, it would result pretty interesting to find a probability distribution function such that the sum of normal variables with zero mean and standard deviation following such unknown distribution could yield a TSP or an ETSP one as a result, though this problem still remains open.

Since both the TSP and the ETSP distributions provide a good fit for daily (log) returns of stock market prices, it is possible that the intraday process somehow could produce these kind of distributions or another type of similar ones, so it is quite interesting to investigate the self-similarity index of such processes, which constitutes the main goal of this paper.

Accordingly, in this section we study the self-similarity exponent of random processes modeled by means of heavy-tailed distributions with underlying ETSP distribution. A similar study carried out for Lévy stable processes can be found in [74].

### Self-similarity index depends on the underlying distribution

Note that at a first glance, one could expect a similar result to the FBM case, namely, that a self-similarity index equal to 0.5 would imply that there is no memory in the series, but in fact, that is not the case. Actually, the results given by Monte Carlo simulation show that the self-similarity index (calculated by means of GHE approach) depends on the distribution, as well as it happens in the LSM case. Next, we analyze how the self-similarity exponent depends on the parameters of the ETSP distribution. To do this, we consider 1024-length time series. Overall, we can state that

- if the underlying distribution of the random process is a TSP (namely, an ETSP for which *λ* = *δ* = 0 and *n* = *m*), then *H* remains close to 0.5, whereas
- if the corresponding distribution is an ETSP (with *λ* > 0), then *H* ≠ 0.5.

**Fig 2:**
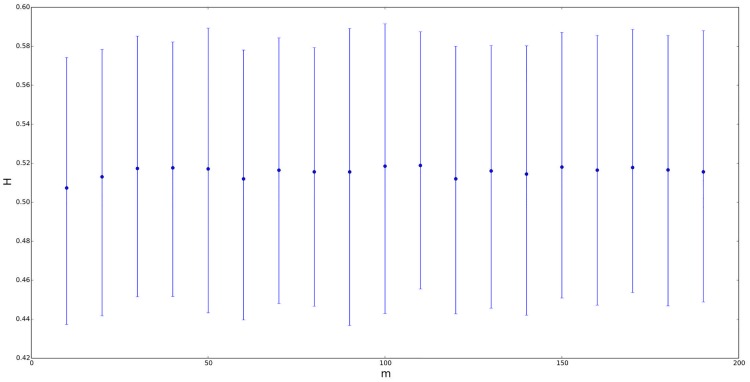
Self-similarity index *H* for symmetric ETSP distributions with parameters *a* = −0.2, *b* = 0.2 vs. *n* = *m* ranging from 10 to 200. The figure above corresponds to *λ* = *δ* = 0, namely, the TSP case. Segments represent the confidence interval of *H* at a 90% confidence level, with the point in the segment being the mean value.

**Fig 3:**
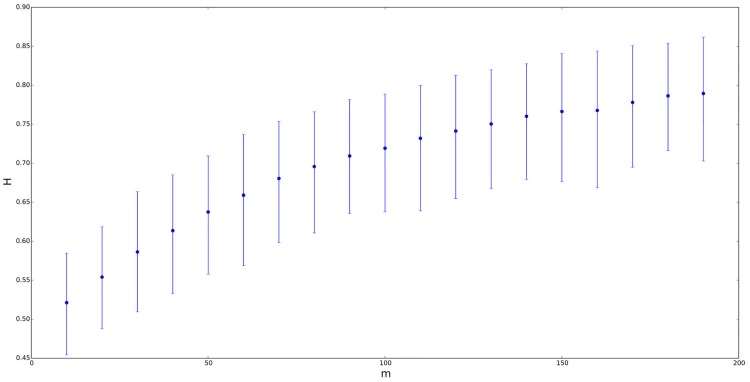
Self-similarity index *H* for symmetric ETSP distributions with parameters *a* = −0.2, *b* = 0.2 vs. *n* = *m* ranging from 10 to 200. The figure above corresponds to *λ* = *δ* = 0.01. Segments represent the confidence interval of *H* at a 90% confidence level, with the point in the segment being the mean value.

**Fig 4:**
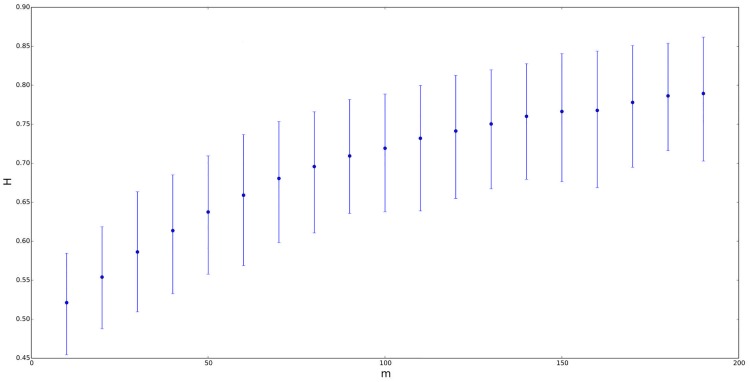
Self-similarity index *H* for symmetric ETSP distributions with parameters *a* = −0.2, *b* = 0.2 vs. *n* = *m* ranging from 10 to 200. The figure above corresponds to *λ* = *δ* = 0.05. Segments represent the confidence interval of *H* at a 90% confidence level, with the point in the segment being the mean value.

**Fig 5:**
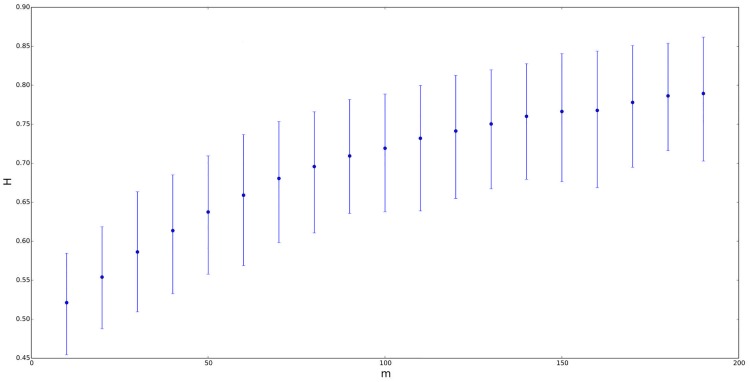
Self-similarity index *H* for symmetric ETSP distributions with parameters *a* = −0.2, *b* = 0.2 vs. *n* = *m* ranging from 10 to 200. The figure above corresponds to *λ* = *δ* = 0.1. Segments represent the confidence interval of *H* at a 90% confidence level, with the point in the segment being the mean value.

Let us provide a more specific analysis about the dependence of *H* on the distribution of the random process. Firstly, to avoid a self-similarity index different from 0.5 due to a mean different from 0, we will work with a symmetric ETSP distribution whose mean is equal to zero and its parameters are *a* = −0.2, *b* = 0.2, *n* = *m* and *λ* = *δ*. Figs 2–5 contain four different situations, where the elevation parameter of the corresponding ETSP distribution varies as follows: *λ* ∈ {0,0.01,0.05,0.1}. In all the cases, we compare the GHE self-similarity estimation vs. *n* = *m* ranging from 10 to 200. Note that for *λ* = *δ* = 0.01, the mean self-similarity exponent goes from 0.5 to 0.8; for *λ* = *δ* = 0.05, *H* ranges from 0.55 to 0.75, and finally, for *λ* = *δ* = 0.1, the Hurst exponent varies from 0.55 to 0.65.

On the other hand, let us also consider a TSP distribution instead of the previous ETSP models for the underlying random process. Thus, if we use *a* = −0.2, *b* = 0.2, *n* = *m* and *λ* = *δ* = 0, then we get that *H* is approximately constant and very close to 0.5 for all *n* = *m*, as we advanced previously (or see both Figs 2 and 3). Similar analysis were also carried out by comparing the self-similarity exponent of ETSP distributions vs. *λ* (instead of vs. *n* = *m*). In this case, we work also with symmetric ETSP distributions (with zero mean) whose parameters are *a* = −0.2, *b* = 0.2, *n* = *m* ∈ {20,100} and *λ* = *δ* ranging from 0.01 to 0.09 by step 0.01. In both cases, the self-similarity exponent of the corresponding time series became more or less stable (close to 0.6 in the case of *m* = 20, and close to 0.7 in the case of *m* = 100).

**Fig 6:**
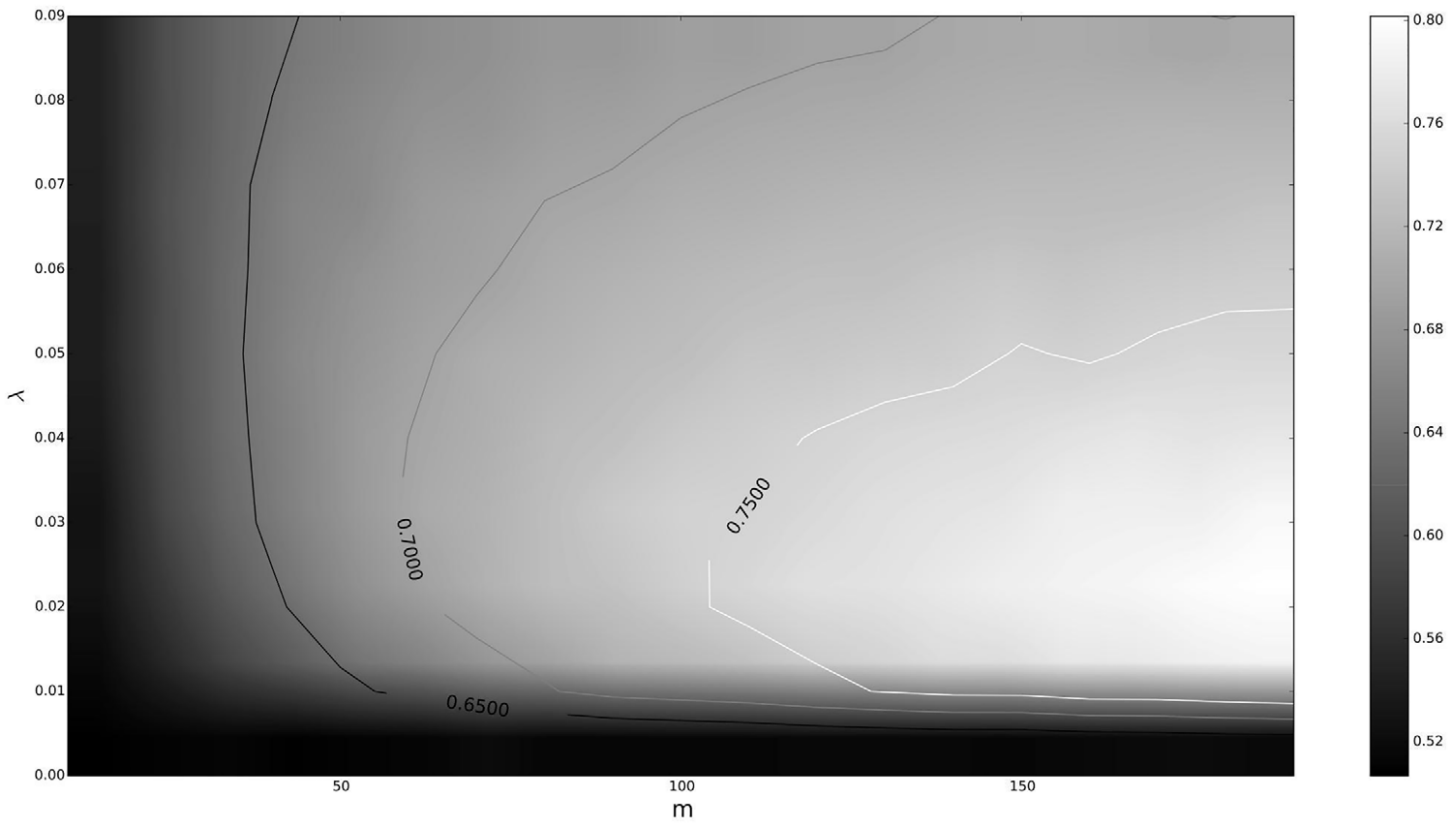
Level curves for the self-similarity exponent *H* of an ETSP distribution. In this case, the parameters were chosen to be *a* = −0.2, *b* = 0.2, and different values of *m* = *n* and *λ* = *δ*.

In Fig 6, it is shown the dependence of *H* on both parameters *m* = *n* and *λ* = *δ*. Note that in this case, the values of *H* range from 0.5 to 0.72.

### Empirical application

In this section, we explore scaling properties for S&P500 stocks as well as for the index itself through analyzing their self-similarity exponents. To do this, let us assume that the distribution of the corresponding series could be properly fitted by means of an ETSP distribution (for daily changes of log returns). Overall, we state that their self-similarity exponents will not provide reliable information about long-term memory properties provided that no additional information about the underlying distribution of such data is previously given. According to that, let us study which stocks from S&P500 index could be properly fitted through an ETSP distribution with explicit parameters given. First of all, let us apply the following notation to refer different self-similarity exponent estimations. Indeed, for a given a stock (or the index itself), let *H*_*Stock*_ denote its self-similarity index, let also *H*_*ETSP*_ denote the self-similarity exponent for an ETSP distribution which properly fits such a stock, and finally, let *H*_*Shuffle*_ denote the self-similarity exponent corresponding to the shuffled time series for that stock. Recall also that the memory component underlying the self-similarity exponent for a given stock could be estimated as *H*_*Stock*_ − *H*_*Shuffle*_. Further, observe that if a time series is shuffled, then its memory component is removed, so if *H*_*Shuffle*_ becomes different from 0.5, then it yields that this is due to the underlying distribution and not to the memory in such series.

Next, let us verify that the stocks from S&P500 index may be properly fitted through an ETSP distribution with explicit parameters. Note that in such a case, one gets that *H*_*Shuffle*_ = *H*_*ETSP*_. In this situation, a result such as *H*_*Stock*_ = *H*_*Shuffle*_ = *H*_*ETSP*_ will throw that there is no memory in the series and that the ETSP distribution becomes a good model for stock (log) returns.

Thus, three statistical test will be carried out in the spirit of bootstrapping (see [60]). The statistic is defined as the GHE exponent *H*. The first of them will consist of checking that *H*_*Shuffle*_ = *H*_*ETSP*_. Accordingly, the null hypothesis is that $\bar{H_{Shuffle}}=\bar{H_{ETSP}}$, where $\bar{H_{Shuffle}}$ denotes the mean of the self-similarity exponents for 500 shuffled series and $\bar{H_{ETSP}}$ denotes the mean for 500 self-similarity exponents corresponding to ETSP samples generated by means of Monte Carlo simulations. The statistical test is given through a two-tailed *p*-value, which is defined as

$$\begin{array}{c} p_{0}=\mathrm{Prob}\;[|\bar{H_{Shuffle}}-\bar{H_{ETSP}}\left|>\right|H_{ETSP}-\bar{H_{ETSP}}\left|\right]. \end{array}$$

On the other hand, the main goal in the second test we perform is to explore persistence in the series, namely, whether *H*_*Stock*_ > *H*_*Shuffle*_. The null hypothesis in this case is $H_{Stock}=\bar{H_{Shuffle}}$, with $\bar{H_{Shuffle}}$ as previously given, while the alternative hypothesis is $H_{Stock}>\bar{H_{Shuffle}}$. The statistical test is given by a one-tailed *p*-value, which is given as *p*_1_ = Prob [*H*_*Stock*_ > *H*_*Shuffle*_].

Moreover, in the third test we want to study anti-persistence in the series, that is, if *H*_*Stock*_ < *H*_*Shuffle*_. The null hypothesis is $H_{Stock}=\bar{H_{Shuffle}}$, whereas the alternative hypothesis is that $H_{Stock}<\bar{H_{Shuffle}}$, where $\bar{H_{Shuffle}}$ is defined as above. The statistical test is given by a one-tailed *p*-value, which is defined as *p*_2_ = Prob [*H*_*Stock*_ < *H*_*Shuffle*_] = 1 − *p*_1_.

Next, let us verify the previous scheme in the actual cases of S&P500 index and its stocks. To do this, we considered data from four years (from March 2011 to April 2014). Firstly, let us test the behavior of S&P500 index. In this way, the optimal fit for the S&P500 index through an ETSP yields the following parameters: *a* = −0.18, *b* = 0.17, *θ* = 0.517, *m* = 25, *n* = 25, *λ* = 0.003 and *δ* = 0.012. Then the self-similarity index for such an ETSP distribution is calculated by means of Monte Carlo simulations. Thus, we find out that the self-similarity exponent of such an ETSP has a mean equal to *H*_*ETSP*_ = 0.59 (with a standard deviation equal to 0.05), whereas the index itself satisfies that *H*_*Stock*_ = 0.49 and the shuffled index series has a mean of *H*_*Shuffle*_ = 0.54 (with a standard deviation equal to 0.03). The ETSP fit for S&P500 index passed the Kolmogorov-Smirnov goodness-of-fit test. Indeed, the three *p*-values for S&P500 index were *p*_0_ = 0.12, so *H*_*Shuffle*_ = *H*_*ETSP*_, *p*_1_ = 0.11 and *p*_2_ = 0.89. Accordingly, we cannot reject any of the null hypothesis of these test provided that a 95% confidence level is considered.

For S&P500 stocks, we find out that only 1.8% of such stocks fail the first test at a confidence level of 95%, so we could conclude that the fitted ETSP distribution does provide a good model for the stocks from the S&P500 index, at least with respect to the self-similarity exponent *H* (note also that the Kolmogorov-Smirnov goodness-of-fit test was also passed by all the stocks). Hence, the ETSP fit seems to be a good model to test for memory. Next, let us summarize and classify the obtained results for memory purposes in S&P500 stocks throughout the selected time period:

- 0.8% of S&P500 stocks failed the second test (for a confidence level of 95%), so we could conclude that any of such stocks present persistent memory.
- 13.4% of S&P500 stocks failed the third test (also for a confidence level of 95%). An example of this case is Praxair Inc. (PX), for which *H*_*Stock*_ = 0.39, *H*_*Shuffle*_ = 0.51 and *H*_*ETSP*_ = 0.56. This yields empirical evidence regarding memory in the series so that the stocks in this case are anti-persistent.
- 85.8% of the stocks gives similar values for all *H*_*Stock*_, *H*_*Shuffle*_ and *H*_*ETSP*_. An example of this situation is O’Reilly Automotive Inc. (ORLY), for which *H*_*Stock*_ = 0.6, *H*_*Shuffle*_ = 0.61 and *H*_*ETSP*_ = 0.61. Accordingly, we could conclude that there is no significant memory in such series, even for self-similarity exponents equal to 0.6.

## Discussion

The main contribution in this paper is to point out that some information about the underlying distribution of a (financial) time series should be previously explored before applying its self-similarity exponent (resp. Hurst exponent) as a reliable tool to test for long-range dependence in such series. This new proposal encourages the readers to be careful to conclude whether there is long-memory in certain stocks (resp. market indices) provided that only their self-similarity exponents (somehow calculated) are considered.

In this way, a common practice is to (implicitly or explicitly) assume that the evolution of a given stock follows a FBM model, and then its self-similarity exponent (resp. its Hurst exponent) is calculated in order to provide some conclusions regarding the existence of memory in such a series. Thus, if the corresponding self-similarity exponent is found to be equal to 0.5, then it is usually concluded that there is no memory in that stock. However, as we state in this paper, if the underlying normal distribution is replaced by a heavy-tailed distribution (ETSP in this case) to model the (log) returns, then a self-similarity index close to 0.5 means nothing about memory. In fact, recall that in the Empirical Application carried out in previous section for S&P500 index and its stocks, we obtained that a number of stocks presented a self-similarity exponent different from 0.5. However, this fact is due to the effect of the underlying distribution and not to the presence of memory in their evolution through time. Thus, in these cases, some reliable information about the underlying distribution in such returns should be previously considered. Indeed, we illustrate this methodology through an application involving all S&P500 stocks as well as the index itself. Overall, we could state that all S&P500 stocks (including the index itself) could be properly fitted by means of an ETSP distribution (since all of them passed both the Kolmogorov-Smirnov goodness-of-fit test as well as the test *H*_*Shuffle*_ = *H*_*ETSP*_). Hence, all the S&P500 stocks were classified into three memory groups: persistent memory stocks, anti-persistent memory stocks and finally, those having no significative memory. Note that only a few stocks presented anti-persistent memory, whereas most of them presented no significative memory and none of them presented persistent memory at the prefixed confidence level (95%).

Finally, we would also like to point out that the approach described in this paper could be also applied in order to validate or reject fits for return distributions of a given (financial) time series. Indeed, though the corresponding fit could pass the Kolmogorov-Smirnov goodness-of-fit test, we encourage the reader to carry out an additional test based on the statistical checking of the equality between *H*_*Shuffle*_ and *H*_*ETSP*_, where the ETSP distribution may be replaced by another distribution.

## References

[1] Cootner P. The Random Character of Stock Market Prices. Cambridge, Mass.: MIT Press; 1964.

[2] Fama EF. The Behavior of Stock-Market Prices. J Bus. 1965;38: 34–105. 10.1086/294743

[3] McDonald JB. Probability distributions for financial models In: Maddala GS, Rao CR, editors. Handbook of statistics, Financial statistics. Amsterdam: Elsevier Science Publishers BV; 1996 pp. 427–461.

[4] Mandelbrot BB. The Variation of Certain Speculative Prices. J Bus. 1963;36: 394–419. 10.1086/294632

[5] Boyarchenko SI, Levendorskii SZ. Option pricing for truncated Lévy processes. Int J Theor Appl Finance. 2000;3: 549–552. 10.1142/S0219024900000541

[6] Carr P, German H, Madan DB, Yor M. The Fine Structure of Asset Returns: An Empirical Investigation. J Bus. 2002;75: 305–332. 10.1086/338705

[7] Kim YS, Rachev ST, Bianchi ML, Fabozzi FJ. Financial market models with Lévy processes and time-varying volatility. J Bank Financ. 2008;32: 1363–1378. 10.1016/j.jbankfin.2007.11.004

[8] Kim YS, Rachev ST, Chung DM, Bianchi ML. The modified tempered stable distribution, GARCH-models and option pricing. Prob Math Stat. 2009;29: 91–117.

[9] Koponen I. Analytic approach to the problem of convergence of truncated Lévy flights towards the Gaussian stochastic process. Phys Rev E. 1995; 52: 1197–1199. 10.1103/PhysRevE.52.1197 9963525

[10] Kozubowski TJ. Geometric stable laws: Estimation and applications. Math Comput Model. 1999;29: 241–253. 10.1016/S0895-7177(99)00107-7

[11] Kozubowski TJ, Panorska AK. Multivariate geometric stable distributions in financial applications. Math Comput Model. 1999;29: 83–92. 10.1016/S0895-7177(99)00107-7

[12] Kozubowski TJ, Podgórski K. Asymmetric Laplace Laws and Modeling Financial Data. Math Comput Model. 2001;34: 1003–1021. 10.1016/S0895-7177(01)00114-5

[13] Hols MCAB, de Vries CG. The limiting distribution of extremal exchange rate returns. J Appl Econometrics. 1991;6: 287–302. 10.1002/jae.3950060306

[14] Hirschberg J, Mazumdar S, Slottje D, Zhang G. Analysing functional forms of stock returns. Appl Financ Econ. 1992;2: 221–227. 10.1080/758527104

[15] Lau HS, Wingender JR, Lau AHL. On Estimating Skewness in Stock Returns. Manage. Sci. 1989;35: 1139–1142. 10.1287/mnsc.35.9.1139

[16] Gray JB, French DW. Empirical comparisons of distributional models for stock index returns. J. Bus. Finan. Account. 1990;17: 451–459. 10.1111/j.1468-5957.1990.tb01197.x

[17] Levy H, Duchin R. Asset Return Distributions and the Investment Horizon. The Journal of Portfolio Management. 2004;30: 47–62. 10.3905/jpm.2004.412319

[18] Praetz PD. The Distribution of Share Price Changes. J. Bus. Res. 1972;45: 49–55. 10.1086/295425

[19] Clark PK. A Subordinated Stochastic Process Model with Finite Variance for Speculative Prices. Econometrica. 1973;41: 135–155. 10.2307/1913889

[20] Greene MT, Fielitz BD. Long-term dependence in common stock returns. J Financ Econ. 1977;4: 339–349. 10.1016/0304-405X(77)90006-X

[21] Hampton J. Rescaled range analysis: Approaches for the financial practitioners, Part 3. Neuro Vest Journal. 1996;4: 27–30.

[22] Lillo F, Farmer JD. The Long Memory of the Efficient Market. Stud Nonlinear Dyn E. 2004;8: 1–19. 10.2202/1558-3708.1226

[23] Panas E. Estimating fractal dimension using stable distributions and exploring long memory through ARFIMA models in Athens Stock Exchange. Appl Financ Econ. 2001;11: 395–402. 10.1080/096031001300313956

[24] Peters EE. R/S Analysis Using Logarithmic Returns. Financ Anal J. 1992;48: 32–37. 10.2469/faj.v48.n6.81

[25] Lo AW. Long-Term Memory in Stock Market Prices. Econometrica. 1991;59: 1279–1313. 10.2307/2938368

[26] Lo AW, MacKinlay AC. Long-term memory in stock market prices In: Dougherty P, ed. A non-random walk down Wall Street. Princeton University Press; 1999.

[27] Sánchez Granero MA, Trinidad Segovia JE, García Pérez J. Some comments on Hurst exponent and the long memory processes on capital markets. Physica A. 2008;387: 5543–5551. 10.1016/j.physa.2008.05.053

[28] Di Matteo T. Multi-scaling in finance. Quant. Financ. 2007;7: 21–36. 10.1080/14697680600969727

[29] Di Matteo T, Aste T, Dacorogna MM. Long-term memories of developed and emerging markets: Using the scaling analysis to characterize their stage of development. J Bank Financ. 2005;29: 827–851. 10.1016/j.jbankfin.2004.08.004

[30] Gençay R, Dacorogna MM, Muller UA, Pictet O, Olsen R. An Introduction to High-Frequency Finance. Academic Press: San Diego; 2001.

[31] Mantegna RN, Stanley HE. Scaling behaviour in the dynamics of an economic index. Nature. 1995;376: 46–49. 10.1038/376046a0

[32] Evertsz CJG. Fractal geometry of financial time series. Fractals-Complex Geom Patterns Scaling Nat Soc. 1995;3: 609–616.

[33] Ghashghaie S, Breymann W, Peinke J, Talkner P, Dodge Y. Turbulent cascades in foreign exchange markets. Nature. 1996;381: 767–770. 10.1038/381767a0

[34] Stanley HE, Mantegna RN. An introduction to econophysics. Cambridge University Press: Cambridge; 2000.

[35] Peters EE. Chaos and Order in the Capital Markets A New View of Cycle, Prices, and Market Volatility. New York: Wiley; 1991.

[36] Peters EE. Fractal Market Analysis: Applying Chaos Theory to Investment and Economics. New York: Wiley; 1994.

[37] Weron A, Weron R. Fractal market hypothesis and two power-laws. Chaos Solitons Fractals. 2000;11: 289–296. 10.1016/S0960-0779(98)00295-1

[38] Kristoufek L. Fractal Markets Hypothesis and the Global Financial Crisis: Scaling, Investment Horizons and Liquidity. Advs Complex Syst. 2012;15: 1250065 10.1142/S0219525912500658

[39] Kristoufek L. Fractal Markets Hypothesis and the Global Financial Crisis: Wavelet Power Evidence. Sci Rep. 2013;3: 2857 10.1038/srep02857 24091386 PMC3790199

[40] Kristoufek L, Vosvrda M. Measuring capital market efficiency: Global and local correlations structure. Physica A. 2013;392: 184–193. 10.1016/j.physa.2012.08.003

[41] Kristoufek L, Vosvrda M. Measuring capital market efficiency: long-term memory, fractal dimension and approximate entropy. Eur Phys J B. 2014;87: 162 10.1140/epjb/e2014-50113-6

[42] Samuelson PA. Proof That Properly Anticipated Prices Fluctuate Randomly. Ind Manag Rev. 1965;6: 41–49.

[43] Lim KP. Ranking market efficiency for stock markets: A nonlinear perspective. Physica A. 2007;376: 445–454. 10.1016/j.physa.2006.10.013

[44] Zunino L, Zanin M, Tabak BM, Pérez DG, Rosso OA. Complexity-entropy causality plane: A useful approach to quantify the stock market inefficiency. Physica A. 2010;389: 1891–1901. 10.1016/j.physa.2010.01.007

[45] Mandelbrot BB, Wallis JR. Robustness of the rescaled range R/S in the measurement of noncyclic long run statistical dependence. Water Resour. Res. 1969;5: 967–988. 10.1029/WR005i005p00967

[46] Weron R. Estimating long range dependence: finite sample properties and confidence intervals. Physica A. 2002;312: 285–299. 10.1016/S0378-4371(02)00961-5

[47] Willinger W, Taqqu MS, Teverovsky V. Stock market prices and long-range dependence. Financ Stoch. 1999;3: 1–13. 10.1007/s007800050049

[48] Kantelhardt JW, Zschiegner SA, Koscielny-Bunde E, Havlin S, Bunde A, Stanley HE. Multifractal detrended fluctuation analysis of nonstationary time series. Physica A. 2002;316: 87–114. 10.1016/S0378-4371(02)01383-3

[49] BenSaïda A. Noisy chaos in intraday financial data: Evidence from the American index. Appl Math Comput. 2014;226: 258–265. 10.1016/j.amc.2013.10.064

[50] Das A, Das P. Does composite index of NYSE represents chaos in the long time scale?. Appl Math Comput. 2006;174: 483–489. 10.1016/j.amc.2005.04.096

[51] Geweke J, Porter-Hudak S. The estimation and application of long memory time series models. J Time Ser Anal. 1983;4: 221–238. 10.1111/j.1467-9892.1983.tb00371.x

[52] Haslett J, Raftery AE. Space-Time Modelling with Long-Memory Dependence: Assessing Ireland’s Wind Power Resource. J R Stat Soc Ser C-Appl Stat. 1989;38: 1–50.

[53] Alessio E, Carbone A, Castelli G, Frappietro V. Second-order moving average and scaling of stochastic time series. Eur Phys J B. 2002;27: 197–200. 10.1007/s10051-002-9020-2

[54] Barabási AL, Vicsek T. Multifractality of self-affine fractals. Phys. Rev. A. 1991;44: 2730–2733. 10.1103/PhysRevA.44.2730 9906256

[55] Trinidad Segovia JE, Fernández-Martínez M, Sánchez-Granero MA. A note on geometric method-based procedures to calculate the Hurst exponent. Physica A. 2012;391: 2209–2214. 10.1016/j.physa.2011.11.044

[56] Sánchez-Granero MA, Fernández-Martínez M, Trinidad Segovia JE. Introducing fractal dimension algorithms to calculate the Hurst exponent of financial time series. Eur Phys J B. 2012;85: 86 10.1140/epjb/e2012-20803-2

[57] Carbone A, Castelli G, Stanley HE. Time-dependent Hurst exponent in financial time series. Physica A. 2004;344: 267–271. 10.1016/j.physa.2004.06.130

[58] Arianos S, Carbone A. Detrending moving average algorithm: A closed-form approximation of the scaling law. Physica A. 2007;382: 9–15. 10.1016/j.physa.2007.02.074

[59] Xu L, Ivanov PC, Hu K, Chen Z, Carbone A, Stanley HE. Quantifying signals with power-law correlations: A comparative study of detrended fluctuation analysis and detrended moving average techniques. Phys Rev E. 2005;71: 051101 10.1103/PhysRevE.71.051101 16089515

[60] Jiang ZQ, Xie WJ, Zhou WX. Testing the weak-form efficiency of the WTI crude oil futures market. Physica A. 2014;405: 235–244. 10.1016/j.physa.2014.02.042

[61] Shao YH, Gu GF, Jiang ZQ, Zhou WX, Sornette D. Comparing the performance of FA, DFA and DMA using different synthetic long-range correlated time series. Sci Rep. 2012;2: 835 10.1038/srep00835 23150785 PMC3495288

[62] Peng CK, Buldyrev SV, Goldberger AL, Havlin S, Sciortino F, Simons M, et al Long-range correlations in nucleotide sequences. Nature. 1992;356: 168–170. 10.1038/356168a0 1301010

[63] Gu GF, Zhou WX. Detrending moving average algorithm for multifractals. Phys Rev E. 2010;82: 011136 10.1103/PhysRevE.82.011136 20866594

[64] Jun WC, Oh G, Kim S. Understanding volatility correlation behavior with a magnitude cross-correlation function. Phys Rev E. 2006;73: 066128 10.1103/PhysRevE.73.066128 16906935

[65] Podobnik B, Stanley HE. Detrended Cross-Correlation Analysis: A New Method for Analyzing Two Nonstationary Time Series. Phys Rev Lett. 2008;100: 084102 10.1103/PhysRevLett.100.084102 18352624

[66] Horvatic D, Stanley HE, Podobnik B. Detrended cross-correlation analysis for non-stationary time series with periodic trends. EPL. 2011;94: 18007 10.1209/0295-5075/94/18007

[67] Zhou WX. Multifractal detrended cross-correlation analysis for two nonstationary signals. Phys. Rev. E. 2008;77: 066211 10.1103/PhysRevE.77.066211 18643354

[68] Jiang ZQ, Zhou WX. Multifractal detrending moving-average cross-correlation analysis. Phys. Rev. E. 2011;84: 016106 10.1103/PhysRevE.84.016106 21867256

[69] Fernández-Martínez M, Sánchez-Granero MA, Trinidad Segovia JE. Measuring the self-similarity exponent in Lévy stable processes of financial time series. Physica A. 2013;392: 5330–5345. 10.1016/j.physa.2013.06.026

[70] Mercik S, Weron K, Burnecki K, Weron A. Enigma of Self-Similarity of Fractional Lévy Stable Motions. Acta Phys Pol B. 2003;34: 3773–3791.

[71] Lamperti JW, Semi-Stable Stochastic Processes. Trans Am Math Soc. 1962;104: 62–78. 10.1090/S0002-9947-1962-0138128-7

[72] Hurst HE. Long-term storage capacity of reservoirs. Trans Am Soc Civ Eng. 1951;6: 770–799.

[73] Mandelbrot BB. Fractals and scaling in finance: discontinuity, concentration, risk. New York: Springer-Verlag; 1997.

[74] Barunik J, Kristoufek L. On Hurst exponent estimation under heavy-tailed distributions. Physica A. 2010;389: 3844–3855. 10.1016/j.physa.2010.05.025

[75] Groenendijk PA, Lucas A, de Vries CG. A Hybrid Joint Moment Ratio Test for Financial Time Series. Discussion paper TI, 98–104/2. 1998;1: 1–38. Available: http://dspace.ubvu.vu.nl/handle/1871/9296.

[76] Peng CK, Buldyrev SV, Havlin S, Simons M, Stanley HE, Goldberger AL. Mosaic organization of DNA nucleotides. Phys Rev E. 1994;49: 1685–1689. 10.1103/PhysRevE.49.1685 9961383

[77] Fernández-Martínez M, Sánchez-Granero MA, Trinidad Segovia JE, Román-Sánchez IM. An accurate algorithm to calculate the Hurst exponent of self-similar processes. Phys Lett A. 2014;378: 2355–2362. 10.1016/j.physleta.2014.06.018

[78] Feder J. Fractals. New York: Plenum Press; 1988.

[79] West BJ, Deering B. The lure of modern science: fractal thinking. Singapore: World Scientific; 1995.

[80] Di Matteo T, Aste T, Dacorogna MM. Scaling behaviors in differently developed markets. Physica A. 2003;324: 183–188. 10.1016/S0378-4371(02)01996-9

[81] Barunik J, Aste T, Di Matteo T, Liu R. Understanding the source of multifractality in financial markets. Physica A. 2012;391: 4234–4251. 10.1016/j.physa.2012.03.037

[82] Morales R, Di Matteo T Aste T. Non-stationary multifractality in stock returns. Physica A. 2013;392: 6470–6483. 10.1016/j.physa.2013.08.037

[83] García CB, García Pérez J, van Dorp JR. Modeling heavy-tailed, skewed and peaked uncertainty phenomena with bounded support. Stat Methods Appt. 2011;20: 463–486. 10.1007/s10260-011-0173-0

[84] van Dorp JR, Kotz S. The Standard Two-Sided Power Distribution and its Properties. With Applications in Financial Engineering. Am Stat. 2002;56: 90–99. 10.1198/000313002317572745

[85] Rachev S, ed. Handbook of Heavy Tailed Distributions in Finance. Amsterdam: Elsevier/North-Holland; 2003.

[86] Linden M. A model for stock return distribution. Int J Financ Econ. 2001;6: 159–169. 10.1002/ijfe.149

[87] Kotz S, van Dorp JR. A link between two-sided power and asymmetric Laplace distributions: with applications to mean and variance approximations. Stat Probab Lett. 2005;71: 382–394. 10.1016/j.spl.2004.11.019

